# Policy development and challenges of global mental health: a systematic review of published studies of national-level mental health policies

**DOI:** 10.1186/s12888-018-1711-1

**Published:** 2018-05-18

**Authors:** Wei Zhou, Yu Yu, Mei Yang, Lizhang Chen, Shuiyuan Xiao

**Affiliations:** 1 Department of Epidemiology and Health Statistics, School of Public Health, Central South University, Changsha, China; 2 Hospital Administration Institute, Xinagya Hospital, Central South University, Changsha, China; 3 Evaluation Department, Xiangya Hospital, Central South University, Changsha, China; 4 Department of Addiction Medicine, Shenzhen Kangning Hospital, Shenzhen Mental Health Center, Shenzhen, China; 5 Department of Social Medicine and Health Management, School of Public Health, Central South University, Changsha, China

**Keywords:** Global mental health, Policy development, Implementation challenges, Systematic review

## Abstract

**Background:**

Mental health policy can be an essential and powerful tool to improve a population’s mental health. However, around one third of countries do not possess a mental health policy, and there are large disparities in population coverage rates between high- and low-income countries. The goal of this study is to identify the transition and implementation challenges of mental health policies in both high-income countries (HICs) as well as middle- and low-income countries (MLICs).

**Methods:**

PubMed, Cochrane Library and Campbell Library were searched from inception to 31 December 2017, for studies on implemented mental health policies at the national level. Abstracts and the main texts of papers were double screened, and extracted data were analysed through thematic synthesis.

**Results:**

A total of 93 papers were included in this study, covering 24 HICs, 28 MLICs and 5 regions. Studies on mental health policies, especially those of MLICs, kept increasing, but MLICs were still underrepresented in terms of publication quantity and study frequency. Based on the included studies, nine policy domains were summarized: service organizing, service provision, service quality, human resources, legislation and human rights, advocacy, administration, surveillance and research, and financing and budgeting. HICs incrementally enriched their policy content in all domains over centuries of development; following HICs’ experience, mental health policies in MLICs have boomed since the 1990s and quickly extended to all domains. Implementation problems in HICs were mainly related to service organizing and service provision; for MLICs, more severe implementation problems converged on financing and budgeting, administration and human resources.

**Conclusions:**

Mental health policy developments in both HICs and MLICs present a process of diversification and enrichment. In terms of implementation, MLICs are faced with more and greater challenges than HICs, especially in funding, human resources and administration. Therefore, future efforts should not only be made on helping MLICs developing mental health policies, but also on promoting policy implementation under MLICs’ local context.

**Electronic supplementary material:**

The online version of this article (10.1186/s12888-018-1711-1) contains supplementary material, which is available to authorized users.

## Background

Mental health policy is a government statement specifying values, principles and objectives for mental health. It can be implemented in the forms of mental health plans, programmes, strategies and legislation at multiple levels [1]. If properly formulated and implemented, mental health policy can be an essential and powerful tool for countries to improve mental health and reduce the burden of mental disorders. Mental health policies have undergone long periods of transition, tracing back to the removal of the chains of lunatics in 1793 by Philippe Pinel, to the second psychiatric revolution in the 1960s thanks to the advent of new psychosocial and biological therapies, to the growth of attention towards mental health policies and finally to a boom of policy statements over the last two decades [2–5]. In 2013, the adoption of the Comprehensive Mental Health Action Plan 2013–2020 by the 66th World Health Assembly would promote further development of mental health policies across the world. Despite the importance of mental health policy, centuries of developments and active promotion by international organizations, by 2011 only 60% of member countries of the World Health Organization (WHO) possessed their own mental health policies, only 71% had mental health plans and just 59% had legislation on mental health. Moreover, large disparities in population coverage rates exist between high- and low-income countries. For example, mental health legislation covers 92% of people living in high-income countries, whereas only 36% are covered in low-income countries [6].

There were international comparative reviews on mental health legislation in the 1950s, 1970s and in 1995 [7–9], as well as reviews of mental health policy in individual countries or certain regions [3, 10–13]. However, these international reviews are mostly outdated and only focused on legislation, and some have a narrow geographic scope. Thus, to update and broaden our understanding on mental health policies, this systematic review aims to present global mental health policy developments and search for the answers to two questions that may be critical to future policy making: (i) What are the focuses of mental health policy content in different countries and at different periods? and (ii) What are the policy implementation problems across the world and throughout the centuries?

To explore these answers, the current review is focused on identifying the differences in mental health policy development trajectories and implementation challenges between high-income countries (HICs) and middle- and low-income countries (MLICs) [14], by using thematic synthesis of published studies of implemented national-level mental health policies. We hope to provide some inspiration to both HICs and MLICs for their future development of mental health policies.

## Methods

### Search strategy and selection criteria

For inclusion in the review we screened published research on implemented mental health policies at the national level. We searched PubMed, the Cochrane Library, and the Campbell Library from their collections’ inception to 31 December 2017. “Policy(ies)”, “national plan”, “national program(me)”, “national strategy(ies)”, “legislation”, “law”, “national reform”, and “system”, were combined with “mental health” in our search. We set no restriction on the publication date or language. A title search was adopted, as the pilot title/abstract search identified a large amount of irrelevant research whose abstracts only mentioned mental health policy in the studies’ background.

Studies were excluded if they: (i) were theoretical studies, describing standards that mental health policies are expected to meet; (ii) focused on only one aspect of policy content (to avoid over-weighted statistics); (iii) studied the process of policy formulation; (iv) were policy interpretation for operation/practice; (v) were news, interviews, editorials, commentaries, letters, personal narratives, speeches, conference abstracts, case reports or research protocols (to guarantee objectivity and research quality).

### Screening, abstraction and synthesis

Identified citations were first screened based on their titles and abstracts, and if they met the selection criteria, their full texts were obtained for further screening to determine whether or not they would be included. The screening process was carried out independently by two authors (ZW and YY), and any disagreement was resolved through consensus.

The authors (ZW, YY, YM, CLZ, and XSY) extracted data on publication year, countries/regions being studied, policy time and content, implementation problems, barriers and progress. Extracted data were compiled by country and further grouped into HICs and MLICs.

As all the included papers except for one were qualitative studies, a thematic synthesis was adopted, which is a tested method for qualitative research in systematic reviews [15] and has been used in health policy and systems research and review [16, 17]. Extracted information was first coded line by line into descriptive themes. Like the process of generating analytical themes in classical thematic synthesis [15, 18], the descriptive themes were then summarized into the main domains of mental health policy. This summarizing process was informed by ideas from the 12 areas of action suggested by the WHO [1].

## Results

**Fig. 1:**
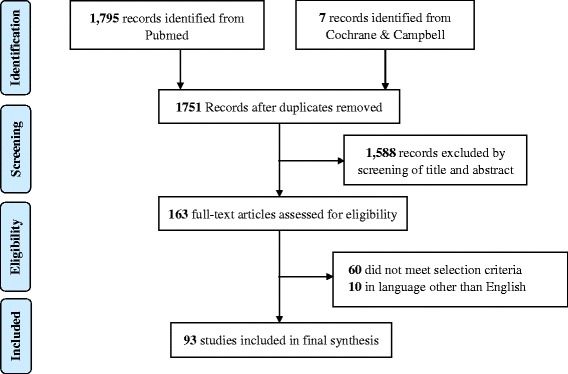
Process and results of study selection

From the initially identified 1751 studies, 1588 references were excluded on the basis of the title or abstract, leaving 163 full-text papers for further scrutiny. Of these, 93 met the selection criteria [3, 4, 8, 10–13, 19–104]. Ten non-English studies were further excluded from analysis (Additional file 1: Appendix A) because their eligibility was difficult for the reviewers to determine (Fig. 1).

### Characteristics of included studies

**Fig. 2:**
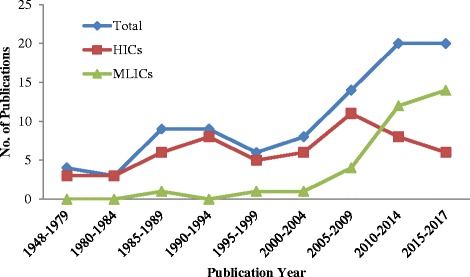
The number of included studies by country categories & publication years

The 93 included papers were published between 1948 and 2017 and covered mental health policies in 24 HICs, 28 MLICs and 5 regions (Additional file 1: Appendix B) on five continents. There was an overall increasing trend in research on mental health policies. Compared to HICs, there was a quick increase of MLICs studies after 2000 (Table 1, Fig. 2).

**Table 1:** The number of included studies by the country category and publication years

Category	1948-1979	1980-1984	1985-1989	1990-1994	1995-1999	2000-2004	2005-2009^a^	2010-2014^b^	2015-2017	Total
HICs	3	3	6	8	5	6	11	8	6	56
MLICs	0	0	1	0	1	1	4	12	14	33
Regions	1	0	2	1	0	1	0	1	0	6
Total	4	3	9	9	6	8	14	20	20	93

^a^One publication studied both HICs and MLICs

^b^One publication studied both HICs and MLICs

Despite the rapid increase of studies on MLICs since 2000, there were only 33 publications included in our review. In terms of frequency, HICs were studied 68 times, while MLICs were studied only 44 times. Among HICs, as well as among all 52 countries, the United States was the most frequently studied (18 times); among MLICs, Ghana was studied with the highest frequency (6 times) (Additional file 1: Appendix B).

### Policy development

Based on the descriptive themes and action areas suggested by the WHO, nine domains were summarized, and their main features are listed as follows: (i) service organizing, referring to the way in which mental health services are organized; (ii) service provision, including promotion, prevention, treatment, rehabilitation, essential drug provision, service availability and accessibility; (iii) service quality, including accreditation and management of service providers, service standards and guidelines; (iv) human resources, including quantity and quality of workforce, professional training and education; (v) legislation and human rights, including the rights of patients in and outside the health sector, social security and welfare; (vi) advocacy, including awareness raising, anti-stigma, empowering consumers; (vii) administration, including coordination within mental health systems and among all levels of governments, designation of agencies’ responsibilities and collaboration across sectors; (viii) surveillance and research, including mental health information systems, monitoring and evaluation of policy implementation and research on service provision; and (ix) financing and budgeting, including government funding arrangements, service payment and health insurance. The detailed list of descriptive themes and their corresponding domains is presented by policy time period and country category in Additional file 1: Appendix C.

**Fig. 3:**
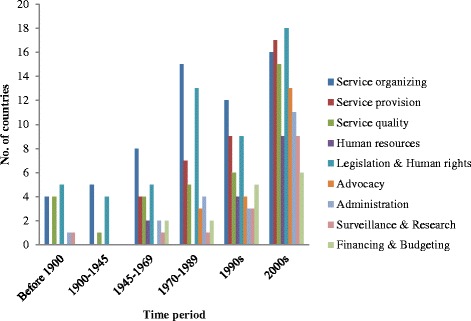
The number of countries expressing each domain in policies based on included studies

Generally, the transition of mental health policy was a process of diversification and enrichment in both HICs and MILCs. The form of mental health policy was diversified from early laws to recent policies, plans, programmes, strategies and legislation, and its content was enriched from the limited focus on institutionalization of patients in asylums to full coverage of all nine policy domains (Fig. 3, Additional file 1: Appendix D). According to the included studies, service organizing, legislation and human rights, service provision and service quality were four domains of traditionally focus across the world.

Differences existed in the development trajectories of HICs and MLICs. Many HICs had a much longer history of mental health policy, dating back to the nineteenth century or even earlier, and their policy content transformed incrementally throughout their practical exploration. Before the end of World War II, institutionalization of service organizing and relevant legislation were the most common policy themes. After that, policy debate in service organizing remained heated, as the movement of transferring patients into the community, originating in North America in the 1960s, spread to almost all HICs in the 1990s. At the same time, measures were also proposed for heightened consideration for patients in service provision and service quality; legislation and human rights extended from protecting patients’ rights in the health sector to guaranteeing patients’ and their families’ social welfare. Since the 1990s, community mental health care has been accepted and developed in almost all HICs, and policy debate in service organizing has become less heated. Currently, policy attention is less focused on certain domains. In comparison, HICs’ policy expressions place less emphasis on financing and budgeting and human resources (Table 2).

**Table 2:** The number of HICs and MLICs expressing each domain in policies based on included studies

Policy domains	HICs						MLICs					
	Before 1900	1900-1944	1945-1969	1970-1989	1990s	2000s	Before 1900	1900-1944	1945-1969	1970-1989	1990s	2000s
Service organizing	3	3	6	11	8	2	1	2	2	4	4	14
Service provision	0	0	3	6	6	3	0	0	1	1	3	14
Service quality	2	0	3	3	4	5	2	1	1	2	2	10
Human resources	0	0	1	0	2	2	0	0	1	0	2	7
Legislation & Human rights	4	3	3	7	6	4	1	1	2	6	3	14
Advocacy	0	0	0	1	1	3	0	0	0	2	3	10
Administration	1	0	2	3	2	3	0	0	0	1	1	8
Surveillance & Research	1	0	1	1	1	2	0	0	0	0	2	7
Financing & Budgeting	0	0	2	1	4	1	0	0	0	1	1	5

Most MLICs have a shorter history of mental health policy and experienced a boom of development in the 1990s and 2000s. As late arrivers, many followed the experience of HICs. Before 1945, mental health policies in countries like India and Algeria had origin in ones in the United Kingdom and France. Between the late 1940s and the 1980s, some MLICs developed their own mental health policies and sporadically discussed service organizing, provision, quality and legislation. In the 1990s, the policy content of MLICs was further enriched, and every policy domain was mentioned by at least one or two countries, but without obvious focuses. In 2000s, the community-oriented policy was accepted as a trend in MLICs; countries like South Africa and Uganda had comprehensive mental health policies covering all nine domains. In comparison, current MLICs policies contain less expression of financing and budgeting, surveillance and research, and human resources (Table 2).

### Implementation challenges

Of the included studies, 64 provided information regarding implementation evaluation [3, 4, 10–13, 19–63, 97–104]. The descriptive themes of implementation problems were categorized into each policy domain, and the unsorted items were grouped by policy time period and country category (Additional file 1: Appendix E).

There were some positive evaluation results. For example, many of the HICs effectively closed down mental hospitals and successfully transferred patients to community-based mental health organizations by the 2000s. Some research showed an increase in treatment access as a result of the implementation of community-oriented policies [28]. However, challenges were also present and problems increased as policy content extended. Slow or inconsistent implementation and under-implementation existed in both HICs and MLICs.

The included studies reported implemented problems of HICs between the 1970s and 1990s mostly on domains of service organizing and service provision, which was closely related to their shift towards community mental health care at that time. However, deinstitutionalization, but without sufficient community mental health care to take over discharged patients, once hampered service availability, accessibility and response to certain populations like the severely ill. In the 2000s, few implementation problems were discussed (Table 3).

**Table 3:** The number of HICs and MLICs with implementation problems in each domain based on included studies

Policy domains	HICs						MLICs					
	Before 1900	1900-1944	1945-1969	1970-1989	1990s	2000s	Before 1900	1900-1944	1945-1969	1970-1989	1990s	2000s
Service organizing	2	1	3	7	5	1	0	0	0	0	0	2
Service provision	0	0	1	6	4	0	0	0	1	0	1	2
Service quality	0	0	3	2	3	0	0	0	0	0	0	1
Human resources	0	0	2	3	2	0	0	0	1	0	2	9
Legislation & Human rights	2	0	2	2	0	0	0	0	0	0	1	3
Advocacy	0	0	1	0	1	0	0	0	1	0	0	6
Administration	0	0	1	5	3	1	0	0	0	2	2	7
Surveillance & Research	0	0	0	2	3	0	0	0	0	0	1	4
Financing & Budgeting	0	0	0	5	2	2	0	0	0	1	4	11
Unsorted	0	0	0	3	3	2	0	0	0	1	6	7

For MLICs, problems have persistently and commonly existed in financing and budgeting, administration and human resources (Table 3). Though some HICs also listed similar problems in these three domains, the problems’ severity was much greater for MLICs. For example, underfunding for many MLICs was so severe that their formulation of mental health policies depended on international funding and the cessation of international funding greatly impacted policy sustainability [44].

## Discussion

### Reflections on policy development

Mental health policies in many MLICs are developed with financial and technical assistance from HICs and international organizations. Thus, they follow the experience and policy practices of HICs. This is reflected in their intensive policy attention on community mental health care in the 2000s and in the comprehensiveness of domain coverage as compared to their high-income counterparts. However, the experience of HICs is accumulated through centuries of development and their current practices are based on well-established mental health systems. Seriously under-resourced and under-developed mental health systems are commonly found in many MLICs [105]. Therefore, MLICs should be cautious about potential problems arising from the direct transplantation of HICs’ experience [106]. Foreign experience developed in different social and cultural contexts should be carefully selected and MLICs should consider policy feasibility based on their own situations. For example, because funding and resources for mental health are scare in MLICs, excessive policy focus will result in fewer inputs in each domain and a possibly result in underdevelopment.

### Reflections on implementation evaluation

Of note is that this review’s findings on policy implementation present only a limited understanding of existing problems, as a lack of information and evaluation of policy implementation itself was listed as a problem in both HICs and MLICs.

Moreover, among the limited implementation information provided by the included studies, many were just general comments such as “while significant gains had been made in mental health reform, these had been uneven across and within jurisdictions” [69]. Even for the included assessment studies, different evaluation frameworks and indicators were applied. The WHO-AIMS (WHO’s Assessment Instrument for Mental Health Systems) Version 2.2 and the WHO Checklist for Mental Health Policy were the only two internationally recognized evaluation instruments related to mental health policies, but they focus on policy content and formulation [107, 108]. Therefore, exploring evaluative frameworks for policy implementation is an important future research topic in mental health policy.

### Limitation

One major limitation is that this review is based on published research rather than first-hand government documents and grey literature. Though advantages of using published research include quick acquisition of policy information from centuries ago, easy determination of a policy’s implementation, and the guarantee of basic quality through a peer review mechanism, there may be bias of result representativeness.

## Conclusion

Global mental health policy developments present a process of diversification and enrichment. The process in HICs is long and incremental through centuries. Based on HICs’ experience, MLICs have quickly developed mental health policies covering domains as comprehensive as HICs, in the recent three decades. In terms of implementation, problems exist in both HICs and MLICs. However, MLICs are faced with more and greater challenges, especially in funding, human resources and administration. Therefore, future efforts should not only be made on helping MLICs developing mental health policies, but also on promoting policy implementation under MLICs’ local context.

## Additional file

Additional file 1: Appendix A to E. (DOC 230 kb)

## Abbreviations

HICs: High-income countries
MLICs: Middle- and low-income countries
WHO: World Health Organization
WHO-AIMS: WHO’s Assessment Instrument for Mental Health Systems
