# Supplementary material for: Policy development and challenges of global mental health: a systematic review of published studies of national-level mental health policies
Source: BMC Psychiatry. 2018 May 18;18:138. doi: 10.1186/s12888-018-1711-1 (PMC5960139; doi:10.1186/s12888-018-1711-1)
Supplement: Supplementary file 1 — Appendix A to E. (DOC 230 kb) [file 12888_2018_1711_MOESM1_ESM.doc]

**Appendix A The list of potentially included studies published in non-English**

1. Bertolote JM. Legislation related to mental health: a review of various international experiences. Rev Saude Publica. 1995 Apr;29(2):152-6. [Article in Portuguese]

2. Bondolfi G1, Müller C. Apropos of the new mental health law in Italy. Schweiz Arch Neurol Psychiatr. 1988;139(1):39-49. [Article in French]

3. Kästner I. The French mental health law of 1938. Psychiatr Neurol Med Psychol (Leipz). 1988 Mar;40(3):145-9. [Article in German]

4. Meyers R. Revision of the Mental Health Law in Australia. Seishin Shinkeigaku Zasshi. 1988;90(3):284-7. [Article in Japanese]

5. ROGINA V. A new British legislation on mental health (Mental Health Act, 1959). Neuropsihijatrija. 1961;9:92-5. [Article in Undetermined Language]

6. Minoletti A1, Zaccaria A. The National Mental Health Plan in Chile: 10 years of experience. Rev Panam Salud Publica. 2005 Oct-Nov;18(4-5):346-58. [Article in Spanish]

7. Borges CF, Baptista TW. The mental health care model in Brazil: a history of policy development from 1990 to 2004. Cad Saude Publica. 2008 Feb;24(2):456-68. [Article in Portuguese]

8. Jaeger M.Changes in French mental health policy. Sante Ment Que. 1995 Spring;20(1):77-87. [Article in French]

9. Marconi J. Policy of mental health in Latin America. Acta Psiquiatr Psicol Am Lat. 1976 Jun;22(2):112-20. [Article in Spanish]

10. Berenzon Gorn S, Saavedra Solano N, Medina-Mora Icaza ME, Aparicio Basaurí V, Galván Reyes J. Evaluation of the mental health system in Mexico: where is it headed? Rev Panam Salud Publica. 2013 Apr;33(4):252-8. [Article in Spanish]

**Appendix B** The list of the studied countries and their frequency

| **Category** | **Frequency**  **(times)** | **Country/Region** |
| --- | --- | --- |
| HICs | 18 | U.S. |
|  | 10 | Australia |
|  | 6 | U.K. |
|  | 5 | Italy |
|  | 4 | Ireland |
|  | 3 | Japan |
|  | 3 | Sweden |
|  | 2 | Poland |
|  | 2 | New Zealand |
|  | 1 | Belgium, Canada ,Czech Republic, Demark, France, Greece, Israel, Korea, Netherlands, Saudi Arabia, Singapore, Slovakia, Spain, Switzerland, United Arab Emirates |
| MILCs | 6 | Ghana |
|  | 3 | South Africa |
|  | 3 | Uganda |
|  | 3 | China |
|  | 3 | India |
|  | 3 | Kenya |
|  | 2 | Zambia |
|  | 1 | Albania, Algeria, Bolivia, Bulgaria, Colombia, Egypt, Hungary, Iraq, Lebanon, Malaysia, Moldova, Montenegro, Mozambique, Nepal, Nigeria, Papua New Guinea, Pakistan, Philippines, Romania, Yugoslav Republic of Macedonia, Zanzibar |
| Regions | 2 | Latin America |
|  | 1 | Eastern Caribbean |
|  | 1 | English-speaking Caribbean |
|  | 1 | Europe in general |
|  | 1 | Global |

**Appendix C** Policy content: the list of descriptive themes and policy domains

| **HICs** | | |
| --- | --- | --- |
| Time period | Descriptive themes | Domains, No. of countries |
| Before 1900 | Institutionalization (Ireland, U.S., Greece) | - Service organizing   (n=3) |
|  | Management of private asylums (Ireland)  Safe treatment (Singapore) | - Service quality   (n=2) |
|  | Humanization of services (Greece)  Procedures for hospitalization  (Ireland, Singapore, New Zealand) | - Legislation & Human rights   (n=4) |
|  | Regionalizing services (Greece) | - Administration   (n=1) |
|  | The monitoring organization (Ireland) | - Surveillance & Research   (n=1) |
| 1900-1944 | Institutionalization (U.S., Japan)  Integrating mental health into health system (New Zealand) | - Service organizing   (n=3) |
|  | Rights protection (Spain)  Guardianship (Japan)  Voluntary hospitalization (New Zealand) | - Legislation & Human rights   (n=3) |
| 1945-1969 | Deinstitutionalization (U.S., Canada, U.K.)  The development of community mental health (U.S., France)  Constructing mental hospitals (Japan, Greece) | - Service organizing   (n=6) |
|  | Comprehensive care/Sectorization (U.S., Poland)  Improving service accessibility (U.S.)  Public education (Japan) | - Service provision   (n=3) |
|  | Management of hospitals and clinical practices (Japan)  Improving (institutional) care quality (U.K., U.S.) | - Service quality   (n=3) |
|  | Professional training (U.S.) | - Human resources   (n=1) |
|  | Guardianship (Japan)  Procedures for involuntary/voluntary hospitalization  (Japan, Ireland, Singapore) | - Legislation & Human rights   (n=3) |
|  | Administrative issues related to district hospitals (Ireland)  Decentralizing government responsibility (Sweden) | - Administration   (n=2) |
|  | Establishing research institutions & supporting research (U.S.) | - Surveillance & Research   (n=1) |
|  | Funding community mental health (U.S.)  Insurance parity between mental and physical health (U.S.)  Payment to district hospitals (Ireland) | - Financing & Budgeting   (n=2) |
| 1970-1989 | Deinstitutionalization (U.S., Denmark, Italy, U.K.)  The development of community mental health  (Greece, Japan, Canada, Italy, Ireland, Netherland, Spain, Sweden, U.K., U.S.)  Integrating mental health into general/primary health  (Greece, Italy, U.K., Spain, Denmark) | - Service organizing   (n=11) |
|  | Improving service accessibility and acceptability (U.S.)  Service continuity (U.S.)  Comprehensive services/ sectorization  (U.S., Ireland, Italy, U.K., Greece, Sweden)  Promotion and prevention (U.S.) | - Service provision   (n=6) |
|  | Improving/assuring service quality (U.S., Sweden)  Service criteria (U.S.)  Improving facilities (Netherland) | - Service quality   (n=3) |
|  | Social security and welfare  (Denmark, Netherland, Sweden, U.S.)  Procedures for involuntary/voluntary hospitalization/treatment (Japan, U.S., Italy, United Arab Emirates)  Rights protection (Japan, U.S., United Arab Emirates) | - Legislation & Human rights   (n=7) |
|  | Involvement of patients and families (Sweden) | - Advocacy   (n=1) |
|  | Regionalizing services/ decentralizing administration  (Greece, Spain)  Coordination/cooperation within mental health system (U.S.)  Intersectoral collaboration (Spain) | - Administration   (n=3) |
|  | Research (U.S.) | - Surveillance & Research   (n=1) |
|  | Service affordability (U.S.)  Funding community mental health (U.S.)  Insurance parity between mental and physical health (U.S.) | - Financing & Budgeting   (n=1) |
| 1990s | Deinstitutionalization (Greece)  The development of community mental health  (Greece, Japan, Australia, Korea, Poland, U.K.)  Integrating mental health into general/primary health  (U.S., Australia, New Zealand) | - Service organizing   (n=8) |
|  | Comprehensive care /Sectorization  (Australia, Poland, U.K., Greece)  Promotion and prevention (Australia, U.K., Italy)  Service continuity (Italy, Australia)  Service accessibility (Italy, New Zealand, Poland, Australia) | - Service provision   (n=6) |
|  | Service standards (Australia, Italy, U.K.)  Improving care quality (Australia, U.K.)  Evidence-based services (U.K.)  Individualized/need-led care (U.K., New Zealand) | - Service quality   (n=4) |
|  | Workforce (Australia)  Support to professionals (U.K.) | - Human resources   (n=2) |
|  | Legislation (Australia, U.K.)  Rights protection  (Australia, Japan, Sweden, U.K., New Zealand)  Social and disability welfare and services  (Australia, Italy, U.S.)  Legal clarification of carers’ responsibilities (Sweden)  Procedures for involuntary hospitalization/treatment  (Japan, Sweden, New Zealand) | - Legislation & Human rights   (n=6) |
|  | Normalization of mental illness (Japan) | - Advocacy   (n=1) |
|  | Coordination within mental health system (Austrlia)  Decentralizing mental health planning (Japan) | - Administration   (n=2) |
|  | Monitoring and evaluating policy implementation (Australia)  Research (Australia) | - Surveillance & Research   (n=1) |
|  | Market-based operation of services (U.S.)  Financing long-term patients (U.S.)  Increasing funding (Australia, Sweden, U.K.) | - Financing & Budgeting   (n=4) |
| 2000s | Deinstitutionalization (Belgium, Czech Republic)  Community mental health (Belgium, Czech Republic) | - Service organizing   (n=2) |
|  | Improving service accessibility (Saudi Arabia, Australia)  Comprehensive services (France, Australia)  Tailored care for minority groups (Australia) | - Service provision   (n=3) |
|  | Accreditation of professionals and facilities  (Saudi Arabia, Australia)  Assuring service quality (Ireland)  Service standards (U.K., Australia)  Improving facilities (U.K.)  Evidence-based services (U.S.)  Need-led care (U.S.) | - Service quality   (n=5) |
|  | Workforce (U.K., Australia) | - Human resources   (n=2) |
|  | Less restrictive care (Saudi Arabia)  Rights protection (Saudi Arabia, Poland, Ireland, Singapore)  Guardianship (Saudi Arabia)  Procedures for involuntary admission (Ireland, Singapore) | - Legislation & Human rights   (n=4) |
|  | Anti-stigma (U.S.)  Social inclusion (Belgium)  Prioritizing mental health (U.K.) | - Advocacy   (n=3) |
|  | Coordination/cooperation within mental health system  (Australia, Belgium, U.S.)  Intersectoral cooperation (Australia) | - Administration   (n=3) |
|  | The information system (U.S.)  Research and assessment (U.S., Slovakia) | - Surveillance & Research   (n=2) |
|  | Guaranteeing funding (Belgium) | - Financing & Budgeting   (n=1) |

| **MLICs** | | |
| --- | --- | --- |
| Time period | Descriptive themes | Domains, No. of countries |
| Before 1900 | Institutionalization (India) | - Service organizing   (n=1) |
|  | Guidelines for establishing asylums (India)  Management of psychiatric institutions (Algeria) | - Service quality   (n=2) |
|  | Procedures for hospitalization (India) | - Legislation & Human rights   (n=1) |
| 1900-1944 | Institutionalization (India, Pakistan) | - Service organizing   (n=2) |
|  | Management of hospitals and workforce (India) | - Service quality   (n=1) |
|  | Procedures for hospitalization (India) | - Legislation & Human rights   (n=1) |
| 1945-1969 | Integrating mental health into general health (India)  Community mental health (Kenya) | - Service organizing   (n=2) |
|  | Prevention (China)  Increasing psychiatric beds (China) | - Service provision   (n=1) |
|  | Recommended clinical practices (China) | - Service quality   (n=1) |
|  | Increasing the number of workforce (China) | - Human resources   (n=1) |
|  | Maintaining social order (China)  Procedures for involuntary hospitalization (Nigeria) | - Legislation & Human rights   (n=2) |
| 1970-1989 | Community mental health/decentralizing mental health services (Papua New Guinea, China, Kenya)  Integrating mental health into general health (Kenya) | - Service organizing   (n=4) |
|  | Service availability and accessibility (India)  Public education (India) | - Service provision   (n=1) |
|  | Management of hospitals (India, Algeria) | - Service quality   (n=2) |
|  | Procedures for voluntary/involuntary treatment  (Ghana, India, Papua New Guinea, Algeria, Kenya)  Judicial system issues (Ghana)  Rights protection (India)  Guardianship (India)  Maintaining social order (China) | - Legislation & Human rights   (n=6) |
|  | Involvement of community (India)  Anti-stigma (Kenya) | - Advocacy   (n=2) |
|  | Establishment of mental health authorities (Kenya) | - Administration   (n=1) |
|  | Free treatment (Ghana) | - Financing & Budgeting   (n=1) |
| 1990s | Community mental health/decentralizing mental health services (Ghana, Zanzibar)  Integrating mental health into primary health (Malaysia, Nepal) | - Service organizing   (n=4) |
|  | Promotion (Ghana, Zanzibar)  Service accessibility (Ghana, Colombia)  Essential medicine list (Ghana)  Prevention and screening (Colombia) | - Service provision   (n=3) |
|  | Improving service quality (Ghana, Colombia) | - Service quality   (n=2) |
|  | The development of human resources (Ghana, Zanzibar) | - Human resources   (n=2) |
|  | Rights protection (India, Albania)  Social welfare (India)  Guardianship (Albania)  Procedures for voluntary/involuntary treatment (Albania)  Legislation (China) | - Legislation & Human rights   (n=3) |
|  | Involvement of patients and families (Ghana)  Anti-discrimination (India)  Social inclusion (India)  The least restrictive care (Albania) | - Advocacy   (n=3) |
|  | Government support and coordination (China) | - Administration   (n=1) |
|  | Monitoring (Ghana, Zanzibar) | - Surveillance & Research   (n=2) |
|  | Financing (Ghana) | - Financing & Budgeting   (n=1) |
| 2000s | Deinstitutionalization (Albania, Moldova)  Community mental health/ decentralizing mental health services  (Albania, Ghana, South Africa, Uganda, Zambia, Hungary, Moldova, Kenya, China, Bolivia, Mozambique, Malaysia)  Integrating mental health into general/primary health  (Albania, Kenya, South Africa, Uganda, Zambia, Ghana, Nigeria, Bolivia)  Equitable service distribution (Uganda)  Cheap and efficient services (Algeria) | - Service organizing   (n=14) |
|  | Service accessibility  (Uganda, Iraq, South Africa, Romania, Mozambique, India)  Prevention and promotion  (Albania, Ghana, Kenya, South Africa, Uganda, Zambia, Romania, China, Nigeria, Montenegro)  Treatment and rehabilitation (China, Nigeria, India)  Comprehensive care (Moldova)  Need-led care (Moldova, Romania)  Special care for vulnerable and disadvantaged groups (Nigeria) | - Service provision   (n=14) |
|  | Evidence-based services/medication (Uganda, Lebanon)  Improving/assuring service quality  (Uganda, Ghana, South Africa, Zambia, Romania)  Capacity building of hospitals (China)  Ethical conduct and integrity of workforce (Uganda)  Service guidelines/standard (China, Egypt, Malaysia, Pakistan)  Management of mental health services (Ghana) | - Service quality   (n=10) |
|  | Training/capacity building of workforce  (Uganda, Albania, Egypt, Ghana, South Africa, Lebanon)  The development of human resources (China)  Support to professionals (Ghana) | - Human resources   (n=7) |
|  | Human rights/rights protection  (Albania, China, South Africa, Zambia, Uganda, India, Iraq, Nigeria, Malaysia, Montenegro, Ghana)  Legislation (China, Kenya, South Africa, Zambia, Algeria)  Guardianship and procedures for voluntary/involuntary treatment (Iraq, Malaysia, Pakistan, Ghana) | - Legislation & Human rights   (n=14) |
|  | Patients/families/community involvement and participation (Albania, Uganda, Kenya)  Advocacy (Uganda, Ghana, South Africa, Zambia, China)  Social inclusion (Albania)  Anti-stigma/discrimination  (Moldova, India, Montenegro, Ghana) | - Advocacy   (n=10) |
|  | National coordination (Ghana)  Coordination/cooperation within mental health system (Uganda)  Intersectoral collaboration  (South Africa, Nigeria, Lebanon, Montenegro)  Establishment of mental health authorities (India, Pakistan) | - Administration   (n=8) |
|  | The information/reporting system  (Uganda, Albania, China, Kenya, South Africa, Zambia, Lebanon)  Research (Uganda, China, Kenya, South Africa, Zambia) | - Surveillance & Research   (n=7) |
|  | Fundraising (Uganda)  Budget (Ghana)  Increasing funding (Albania)  Financing (Ghana, South Africa, Uganda, Zambia) | - Financing & Budgeting   (n=5) |

| **Regions** | | |
| --- | --- | --- |
| Time period | Descriptive themes | Domains, No. of regions |
| Before 1900 | -- | -- |
| 1900-1944 | -- | -- |
| 1945-1969 | Sectorisation (Europe in general ) | - Service provision   (n=1) |
|  | Procedures for hospitalization (Global)  Rights protection (Global) | - Legislation & Human rights   (n=1) |
| 1970-1989 | Community mental health (Europe in general, Global)  Integrating mental health into general health  (English-speaking Caribbean) | - Service organizing   (n=3) |
|  | Comprehensive services (Global) | - Service provision   (n=1) |
|  | Accreditation of facilities (Eastern Caribbean)  Better services (Europe in general) | - Service quality   (n=2) |
|  | Revising legislation (English-speaking Caribbean)  Procedures for hospitalization and criminal issues  (Eastern Caribbean)  Rights protection (Global) | - Legislation & Human rights   (n=3) |
| 1990s | Community mental health/ decentralizing mental health services (Latin America)  Integrating mental health into general/primary health  (Latin America) | - Service organizing   (n=1) |
|  | Service accessibility (Latin America)  Comprehensive services (Latin America) | - Service provision   (n=1) |
|  | Service continuity (Latin America)  Prevention and promotion (Latin America) | - Service quality   (n=1) |
|  | Workforce training (Latin America) | - Human resources   (n=1) |
|  | Legislation and rights protection (Latin America) | - Legislation & Human rights   (n=1) |
| 2000s | Community mental health (English-speaking Caribbean)  Integrating mental health into general/primary health  (English-speaking Caribbean) | - Service organizing   (n=1) |

**Appendix D The number of countries expressing each domain in policies in included studies**

| Policy domains | Before 1900 | 1900-1945 | 1945-1969 | 1970-1989 | 1990s | 2000s |
| --- | --- | --- | --- | --- | --- | --- |
| Service organizing | 4 | 5 | 8 | 15 | 12 | 16 |
| Service provision | 0 | 0 | 4 | 7 | 9 | 17 |
| Service quality | 4 | 1 | 4 | 5 | 6 | 15 |
| Human resources | 0 | 0 | 2 | 0 | 4 | 9 |
| Legislation & Human rights | 5 | 4 | 5 | 13 | 9 | 18 |
| Advocacy | 0 | 0 | 0 | 3 | 4 | 13 |
| Administration | 1 | 0 | 2 | 4 | 3 | 11 |
| Surveillance & Research | 1 | 0 | 1 | 1 | 3 | 9 |
| Financing & Budgeting | 0 | 0 | 2 | 2 | 5 | 6 |

**Appendix E Implementation problems**: the list of descriptive themes and policy domains

| **HICs** | | |
| --- | --- | --- |
| Time period | Descriptive themes | Domains, No. of countries |
| Before 1900 | Untreated patients (Ireland)  Incomprehensive services (Greece) | - Service provision   (n=2) |
|  | Poorly implementing humanization of services (Greece)  Failed in establishing welfare system (Greece)  Misuse of legislation (Ireland) | - Legislation & Human rights   (n=2) |
| 1900-1944 | Poorly implementing institutionalization/building hospitals (Japan) | - Service organizing   (n=1) |
| 1945-1969 | Failed in deinstitutionalization/ Neoinstitutionalization (U.S.)  Slow development of community mental health (U.S., Canada)  Poorly implementing institutionalization/building hospitals  (Japan) | - Service organizing   (n=3) |
|  | Hyperspecializaiton/inability of comprehensive care (France) | - Service provision   (n=1) |
|  | Inability of individualized care (U.K.)  Inconsistent community services (U.S.)  Low psychiatric sophistication and custodial nature of hospitals (Japan) | - Service quality   (n=3) |
|  | Decrease/shortage of workforce quantity (U.K., U.S.)  Unequal distribution of workforce (U.S.) | - Human resources   (n=2) |
|  | Unsuccessful legislation (U.K.)  Relatively permanent hospitalization (Japn) | - Legislation & Human rights   (n=2) |
|  | Repression to community participation/involvement (U.S.) | - Advocacy   (n=1) |
|  | Low autonomy of community services (U.S.)  No/poor coordination within mental health system (U.S.) | - Administration   (n=1) |
| 1970-1989 | Slow/poor development of community mental health  (Denmark, U.S., Italy, Greece, Japan, Ireland)  Slow deinstitutionalization (Italy, Netherland) | - Service organizing   (n=7) |
|  | Unmet needs of the acutely/severely/chronically ill  (Italy, Spain, Sweden, U.S.)  Low service availability/accessibility (Spain)  Unequal distribution of services (U.K.)  Inability of comprehensive care (Sweden, Greece)  Inconsistent community services (U.S.) | - Service provision   (n=6) |
|  | Poor effectiveness of care (Italy)  Inconsistent service quality (U.K,) | - Service quality   (n=2) |
|  | Overloaded workforce (U.K.)  Incompetent workforce (U.K., Italy, Greece) | - Human resources   (n=3) |
|  | Homelessness (U.S.)  Unmet needs of social welfare/assistance to patients (U.S.)  Unworkable patient review system (Japan) | - Legislation & Human rights   (n=2) |
|  | No/poor coordination within mental health system  (Italy, U.S., Spain, Greece)  No/poor intersectoral collaboration (Spain, Sweden)  Low government motivation (Spain)  No/poor coordination of policy implementation (U.S., Greece)  Poor clarity of government/agency responsibilities (U.S.) | - Administration   (n=5) |
|  | No/poor electronic information system (Italy)  No information of policy implementation (Greece) | - Surveillance & Research   (n=2) |
|  | More funding competition between facilities (Italy, Sweden)  Underfunding (Spain, Japan, Italy)  Poor budgeting (Spain, Japan, Italy, U.S.) | - Financing & Budgeting   (n=5) |
|  | Inconsistent policy implementation (Italy)  Inconsistently achieving policy objectives (Spain)  Under-implementation of policy (Italy)  Over-complexity/poor-clarity of policy (U.S., Italy) | - Unsorted   (n=3) |
| 1990s | Slow/poor development of community mental health  (Korea, Poland)  Slow integration of mental health into general health (Australia)  Poor deinstitutionalization (Greece, U.K.) | - Service organizing   (n=5) |
|  | Unequal distribution of facilities (Poland)  Low/Inconsistent (inpatient) service availability/accessibility  (Sweden, U.K., Italy)  Unmet needs of certain populations (U.K) | - Service provision   (n=4) |
|  | Inconsistent service quality (Poland, U.S.)  Poor physical facilities (U.K.) | - Service quality   (n=3) |
|  | Unequal distribution of workforce (Poland)  Shortage of workforce (U.K.) | - Human resources   (n=2) |
|  | Marginalizing and discriminating patients (Australia)  Poor community involvement (Australia) | - Advocacy   (n=1) |
|  | Poor clarity of government/agency responsibilities (Sweden)  No/poor intersectoral collaboration (U.K.)  Poor management capacity (U.K.)  Poor coordination mechanism (Australia) | - Administration   (n=3) |
|  | No/poor electronic information system (Italy, U.K.)  Lacking policy evaluation (U.S.) | - Surveillance & Research   (n=3) |
|  | Underfunding (U.K.)  Poor budgeting (U.S.) | - Financing & Budgeting   (n=2) |
|  | Inconsistent policy implementation (U.K.)  Slow policy implementation (Israel)  Under-implementation of policy (U.K.)  Poor clarity of policy (U.S., U.K.) | - Unsorted   (n=3) |
| 2000s | Inconsistent community mental health (Italy) | - Service organizing   (n=1) |
|  | Insufficient policy leadership (Australia) | - Administration   (n=1) |
|  | Underfunding (Canada, Australia)  Poor budgeting (Australia) | - Financing & Budgeting   (n=2) |
|  | Inconsistent implementation (Australia, United Arab Emirates) | - Unsorted   (n=2) |

| **MLICs** | | |
| --- | --- | --- |
| Time period | Descriptive themes | Domains |
| Before 1900 | -- | -- |
| 1900-1944 | -- | -- |
| 1945-1969 | Unmet needs of the severely/chronically ill (China) | - Service provision   (n=1) |
|  | Incompetent workforce (China) | - Human resources   (n=1) |
|  | Discrimination against patients (China) | - Advocacy   (n=1) |
| 1970-1989 | Lacking translation policy into implementation plans (China)  Partial enactments of law implementation (Algeria) | - Administration   (n=2) |
|  | Unaffordable services (China) | - Financing & Budgeting   (n=1) |
|  | Poor clarity of policy (Algeria) | - Unsorted   (n=1) |
| 1990s | Lacking services and medicine (Nepal) | - Service provision   (n=1) |
|  | Incompetent workforce (China)  Shortage of workforce (Nepal) | - Human resources   (n=2) |
|  | Lacking legislation (Nepal) | - Legislation & Human rights   (n=1) |
|  | Low government motivation (China)  Lacking stakeholders’ involvement (Nepal) | - Administration   (n=2) |
|  | Poor recording system (Nepal) | - Surveillance & Research   (n=1) |
|  | Underfunding (China, Colombia, Kenya, Nepal) | - Financing & Budgeting   (n=4) |
|  | Inconsistent policy implementation (Ghana, South Africa)  Slow policy implementation (Albania)  Inappropriate context for policy implementation (Albania)  Under-implementation (Nigeria, Kenya, Nepal) | - Unsorted   (n=6) |
| 2000s | Minimal reduction in deinstitutionalization (Bolivia)  Failure in decentralization (Mozambique) | - Service organizing   (n=2) |
|  | Poor service availability/accessibility (Egypt, Ghana)  Unmet needs of uncertain populations (Egypt) | - Service provision   (n=2) |
|  | Poor service quality (Ghana) | - Service quality   (n=1) |
|  | Shortage of workforce  (Egypt, Ghana, Zanzibar, Nigeria, Bolivia, Mozambique)  Incompetent workforce  (Ghana, South Africa, Uganda, Zambia, Nigeria) | - Human resources   (n=9) |
|  | Limited legislative authority (Philippines)  Outdated legislation and slow progress of renewing (Nigeria)  Inadequate legal infrastructure (Ghana) | - Legislation & Human rights   (n=3) |
|  | Poor dissemination/communication of policy  (Ghana, South Africa, Uganda, Zambia)  Stigma and discrimination associated with mental disorders (Nigeria, Bolivia) | - Advocacy   (n=6) |
|  | Lacking translation policy into implementation plans  (Ghana, South Africa, Uganda, Zambia)  No/poor coordination of policy implementation  (Ghana, South Africa, Uganda, Zambia, Egypt, Nigeria)  No/poor coordination within mental health system (Egypt)  Low government motivation/priority (Ghana, Nigeria, Bolivia)  Low autonomy of local levels within health system  (Ghana, South Africa, Uganda, Zambia) | - Administration   (n=7) |
|  | Lacking monitoring on policy implementation  (Bulgaria, South Africa)  Lacking surveillance for mental health (Nigeria, Bolivia) | - Surveillance & Research   (n=4) |
|  | Underfunding  (Ghana, South Africa, Uganda, Zambia, Hungary, Moldova, Egypt, Philippines, Zanzibar, Bolivia, Lebanon)  Poor budgeting  (Ghana, South Africa, Uganda, Zambia, Hungary, Moldova, Egypt, Philippines, Zanzibar) | - Financing & Budgeting   (n=11) |
|  | Inconsistent policy implementation (Ghana)  Under-implementation of policy  (Uganda, Zambia, Ghana, Pakistan)  Poor clarity of policy (Ghana, South Africa, Uganda, Zambia)  Poor feasibility of policy  (Ghana, South Africa, Uganda, Zambia)  Unmet policy objectives (Egypt, Algeria)  Slow policy implementation (Algeria, Pakistan) | - Unsorted   (n=7) |

| **Regions** | | |
| --- | --- | --- |
| Time period | Descriptive themes | Domains |
| 19^th^ century or earlier | -- | -- |
| Early 20^th^ century-WWII | -- | -- |
| 1945-1960s | -- | -- |
| 1970s-1980s | -- | -- |
| 1990s | Failed in deinstitutionalization  (English-speaking Caribbean)  Poor integration of mental health into primary health  (English-speaking Caribbean) | - Service organizing   (n=1) |
|  | Shortage of workforce (English-speaking Caribbean) | - Human resources   (n=1) |
|  | Low government motivation (Latin America) | - Administration   (n=1) |
|  | Underfunding (Latin America) | - Financing & Budgeting   (n=1) |
|  | Inconsistent policy implementation (Latin America)  Under-implementation of policy  (English-speaking Caribbean)  Social/culture obstacles for policy implementation  (Latin America) | - Unsorted   (n=2) |
| 2000s | -- | -- |
